# Stress-associated transient enlargement of pre-existing adrenal hyperplasia in 21-hydroxylase deficiency: a case report

**DOI:** 10.3389/fendo.2026.1878903

**Published:** 2026-07-10

**Authors:** Chengcheng Zheng, Lianling Zhao, Tao Chen

**Affiliations:** Department of Endocrinology and Metabolism, West China Hospital, Sichuan University, Chengdu, Sichuan, China

**Keywords:** 21-hydroxylase deficiency, adrenal enlargement, adrenal reserve, case report, congenital adrenal hyperplasia, stress response

## Abstract

**Background:**

The adrenal gland plays a pivotal role in the stress response via the hypothalamic–pituitary–adrenal (HPA) axis and the sympatho–adrenomedullary system (SAMS). Congenital adrenal hyperplasia (CAH) results from pathogenic variants in genes encoding adrenal steroidogenic enzymes. The most common form, 21-hydroxylase deficiency, impairs cortisol biosynthesis, leading to compensatory overproduction of adrenocorticotropic hormone (ACTH) and chronic adrenal hyperplasia.

**Case presentation:**

A 40-year-old man was referred for incidentally detected bilateral adrenal lesions on abdominal computed tomography (CT). CT revealed marked bilateral adrenal enlargement with clear contours. One month before admission, he had an acute upper respiratory tract infection and recovered after treatment with a cephalosporin. On admission, physical examination showed hyperpigmentation of the nipples region, lips, gums, and buccal mucosa. Superficial lymph nodes were palpable. He reported a history of infertility and suspected precocious puberty. Endocrinology markers demonstrated an elevated ACTH level (180 ng/L; reference range 5.0–78.0 ng/L) with a cortisol level of 174 nmol/L (reference range 133.0–537.0 nmol/L). Luteinizing hormone and follicle-stimulating hormone levels were both low, whereas dehydroepiandrosterone sulfate was elevated. Markedly elevated 17α-hydroxyprogesterone levels and a blunted cortisol response were observed under ACTH-stimulated conditions. Based on the clinical phenotype and biochemical findings, CAH due to 21-hydroxylase deficiency was diagnosed and considered consistent with the simple virilizing form, which was subsequently genetically supported by the CYP21A2 I172N homozygous mutation in exon 4. Remarkably, without any specific treatment, adrenal volume decreased substantially within one month, from 61.06 cm^3^ to 33.47 cm^3^, corresponding to an approximately 1.8-fold reduction, although the adrenal glands remained enlarged compared with normal reference values. After excluding adrenal-related tumors, hemorrhage, infections and autoimmune diseases, we postulated that the transient morphological changes reflected stress-induced compensatory hypertrophy and/or hyperplasia of the adrenal cortex following the respiratory infection, aimed at augmenting cortisol production to modulate inflammation and maintain homeostasis.

**Conclusions:**

In patients with 21-OHD and residual enzymatic activity, infectious stress may trigger transient enlargement of pre-existing adrenal hyperplasia, potentially as a compensatory response to increased cortisol demand, followed by partial regression after stress resolution.

## Introduction

Congenital adrenal hyperplasia (CAH) is a group of autosomal recessive disorders caused by pathogenic variation in genes encoding adrenal steroidogenic enzymes. More than 95% cases of CAH are attributable to 21-hydroxylase deficiency (21-OHD) due to mutations in the CYP21A2 gene (1). Disease phenotypes generally form a continuum that correlates with CYP21A2 genotypes and the expected residual 21-hydroxylase activity associated with each genotype (2, 3). Adrenal insufficiency occurs when the adrenal glands cannot produce enough cortisol, leading to loss of negative feedback on the hypothalamic-pituitary axis and counter-regulatory overproduction of adrenocorticotropic hormone (ACTH), which in turn drives excessive adrenal androgen production and adrenal hyperplasia. As a pivotal organ in the stress response, the adrenal gland secretes cortisol from the adrenal cortex and epinephrine from the adrenal medulla via the hypothalamic-pituitary-adrenal (HPA) axis and the sympatho–adrenomedullary system (SAMS), respectively. The zona fasciculata (ZF), which account for more than 70% of the adrenal cortex, is the primary site of cortisol production (4). Several studies have reported that various stressors—such as septic shock (5), major depression (6, 7), obstructive sleep apnea hypopnea syndrome (8), bulimia nervosa (9), and other stressful conditions (10, 11)—may induce adrenal morphological and functional changes, resulting in adrenal enlargement and supraphysiological cortisol secretion. Although adrenal enlargement and altered adrenal morphology have been described in patients with CAH, and adrenal volume has been associated with hormonal control in 21-OHD, prior studies have mainly focused on chronic adrenal morphology rather than short-term stress-related adrenal volume fluctuation (12, 13). To our knowledge, transient enlargement of pre-existing adrenal hyperplasia temporally associated with an acute infectious stressor has not been well documented in CAH patients.

Herein, we present a case of CAH in which an acute upper respiratory tract infection appeared to induce marked bilateral adrenal enlargement. The enlarged adrenal glands subsequently showed substantial partial regression within one month, with an approximately 1.8-fold reduction in volume, without any specific treatment. Notably, as bilateral adrenal glands gradually decreased in size, cortisol levels declined and ACTH levels increased, prompting initiation of hydrocortisone therapy. We present the following case in accordance with the CARE reporting checklist.

## Case description

A 40-year-old man developed myalgia, gingival swelling, fever, and fatigue after staying up late for three consecutive days. He was diagnosed with an acute upper respiratory tract infection, supported by increased leukocyte of 14600/μL (reference range: 3500-9500/μL) and neutrophil of 9600/μL (1800-6300/μL) on blood testing and inflammatory changes on lung computed tomography (CT). His symptoms completely resolved after three days of cephalosporin therapy. During hospitalization, abdominal ultrasonography, performed as part of the routine inpatient evaluation, revealed markedly heterogeneous echogenic mass-like lesions in the bilateral adrenal regions. Given this unexpected finding, contrast-enhanced abdominal CT was subsequently performed for further characterization, which demonstrated marked bilateral adrenal enlargement with well-defined margins (**Figure 1**). Morning serum cortisol was 372 nmol/L (133–537 nmol/L), and adrenocorticotropic hormone (ACTH) was 44.9 ng/L (5.0-78.0 ng/L). The renin-angiotensin-aldosterone system (RAAS) was activated, with elevated plasma aldosterone at 30.2 ng/dL (9.8-27.5 ng/dL) and plasma renin activity exceeding 12.0 ng/mL/h (0.9-6.6 ng/mL/h). Plasma norepinephrine and epinephrine levels were within the normal range. He was subsequently admitted to our institution three and a half weeks later.

**Figure 1:**
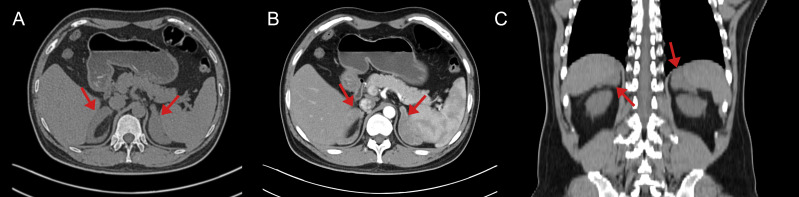
CT of the adrenal glands during infectious stress. **(A)** Axial non-contrast CT. **(B)** Axial contrast-enhanced CT. **(C)** Sagittal reformatted CT. Red arrows indicate bilateral adrenal enlargement.

On admission, his height was 154.0 cm (father 165.0 cm, mother 151.0 cm). Notably, his growth peaked at 11 years old, and his height had not changed since age 15. His blood pressure, heart rate, and body temperature were 108/73 mmHg, 76 beats/min, and 36.1 °C, respectively. Hyperpigmentation was noted in the nipples area, lips, gums, and buccal mucosa. Superficial lymph nodes were palpable in bilateral anterior cervical, submandibular, axillary, and inguinal regions. Cardiac and pulmonary auscultation was unremarkable, and abdominal examination revealed no abnormalities. He had no genital atrophy or defluxion of pubic hair. His medical history was notable for secondary sterility. He had a 16-year-old daughter from a previous marriage, but he and his current wife had been unable to conceive during 8 years of marriage, and he was found to have decreased sperm motility. He denied a history of chronic disease. His parents and younger brother were healthy, and there was no significant family history.

Routine blood tests showed normal serum electrolytes and normal renal and liver function, but abnormal lipids profile, with elevated triglyceride level (1.87 mmol/L) and low high-density lipoprotein cholesterol (0.8 mmol/L). **Table 1** summarizes the clinical parameters on admission. Endocrine evaluation revealed markedly elevated 17α-hydroxyprogesterone (17-OHP) at 157.4 ng/mL (0.5-2.1 ng/mL), increased dehydroepiandrosterone sulfate (DHEA-S) at 12.6 μmol/L (2.41-11.60 μmol/L) and progesterone at 5.33 ng/mL (< 0.149 ng/mL). In contrast, luteinizing hormone (LH) and follicle-stimulating hormone (FSH) were both below the assay detection limit of 0.3 IU/L (1.7-8.6 IU/L and 1.5-12.4 IU/L, respectively). Serum cortisol at 08: 00 was 174 nmol/L (133–537 nmol/L) and ACTH was 180 ng/L (5.0-78.0 ng/L). Given the marked bilateral adrenal enlargement and the normal serum cortisol and ACTH levels measured at the local hospital, 24-hour urinary free cortisol was assessed to characterize endogenous cortisol secretion. The 24-hour urinary free cortisol level was 121.3 ug/24 h (20.3-127.6 ug/24 h) with a urine volume of 2.4 L. Subsequently, an ACTH stimulation test was performed to evaluate adrenal cortical reserve and the adequacy of the cortisol response under stimulated conditions. After intravenous administration of 250 μg of synthetic ACTH (Shanghai First Bio-chemical Pharmaceutical Company, H31022101) over 30 min, cortisol showed a blunted response, increasing only from 128 nmol/L at baseline to 189 nmol/L at 120 min. Meanwhile, 17-OHP remained markedly elevated throughout the test and peaked at 130.7 ng/mL at 60 min, suggesting a substantial impairment of 21-hydroxylase activity and supporting the diagnosis of 21-OHD (**Table 2**).

**Table 1:** Clinical biochemical and hormonal parameters on admission in the patient.

Peripheral blood			Blood biochemistry			Endocrine markers		
Red blood cells	464	430-580 × 10^4^/μL	TB	18.5	5.0-28.0 umol/L	PRL	21.4	4.6-21.4 ng/mL
Hemoglobin	149	130–175 g/L	DB	4.2	< 8.8 umol/L	SHBG	12.2	18.3-54.1 nmol/L
Platelet count	18.3	10.0-30.0 × 10^4^/μL	IDB	14.3	< 20.0 umol/L	Free testosterone	109.8	45.4-142.5 pg/mL
White blood cells	5200	3500-9500/μL	ALT	49	< 50 IU/L	DHEA-S	12.6	2.41-11.6 umol/L
Neutrophils	2610	1800-6300/μL	AST	30	< 40 IU/L	LH	< 0.3	1.7-8.6 IU/L
Lymphocytes	2060	1100-3200/μL	Total protein	71.2	65.0-85.0 g/L	FSH	< 0.3	1.5-12.4 IU/L
Monocytes	390	100-600/μL	Albumin	48.7	40.0-55.0 g/L	Estradiol	17.2	11.3-43.2 pg/mL
Eosinophils	100	20-520/μL	Creatinine	79	68–108 umol/L	Progesterone	5.33	<0.15 ng/mL
Basophils	40	0-60/μL	BUN	4.2	3.1-8.0 mmol/L	17-OHP	157.4	0.5-2.1 ng/mL
			UA	464	240–490 umol/L	Renin	9.35	0.93-6.56 ng/mL/h
Electrolytes			Plasma glucose	4.43	3.90-5.90 mmol/L	Angiotensin II	80.3	55.3-115.3 ng/L
Sodium	139.1	137.0-147.0 mmol/L	ALP	113	51–160 IU/L	Aldosterone	41.8	9.8-27.5 ng/dL
Potassium	4.15	3.50-5.30 mmol/L	LDH	145	120–250 IU/L	cortisol	174	133–537 nmol/L
Chloride	101.9	99.0-110.0 mmol/L	Triglyceride	1.87	0.29-1.83 mmol/L	ACTH	180	5–78 ng/L
Calcium	2.36	2.11-2.52 mmol/L	Cholesterol	4.02	2.80-5.70 mmol/L	Adrenaline	53	54–122 ng/L
Magnesium	0.88	0.75-1.02 mmol/L	HDL-C	0.80	> 0.9 mmol/L	Noradrenaline	589	272–559 ng/L
Phosphorus	1.09	0.85-1.51 mmol/L	LDL-C	2.62	< 4.0 mmol/L	UFC	124.8	20.3-127.6 ug/24 h

17-OHP, 17α-hydroxyprogesterone; ACTH, adrenocorticotropic hormone; ALP, alkaline phosphatase; ALT, alanine aminotransferase; AST, aspartate aminotransferase; BUN, blood urea nitrogen; DB, direct bilirubin; DHEA-S, dehydroepiandrosterone sulfate; FSH, follicle-stimulating hormone; HDL-C, high-density lipoprotein cholesterol; IDB, indirect bilirubin; LDH, lactate dehydrogenase; LDL-C, low-density lipoprotein cholesterol; LH, luteinizing hormone; PRL, prolactin; SHBG, sex hormone–binding globulin; TB, total bilirubin; UA, uric acid; UFC, urinary free cortisol.

**Table 2:** Results of the ACTH stimulation test.

ACTH stimulation test	-15 min	0 min	30 min	60 min	90 min	120 min
Cortisol (nmol/L)	152	128	135	162	176	189
17-OHP (ng/mL)	55.2	37.62	100.94	130.7	94.6	56.4

17-OHP, 17α-hydroxyprogesterone; ACTH, adrenocorticotropic hormone.

Ultrasonography of the superficial lymph nodes showed multiple enlarged lymph nodes in the bilateral cervical, supraclavicular, axillary, and inguinal regions. The largest was located in the submandibular region, measuring approximately 20 × 7mm, with an unclear boundary between the cortex and medulla. Abdominal CT demonstrated bilateral adrenal enlargement with well-defined margins, while the degree of adrenal enlargement decreased substantially over one month, with adrenal volume declining from 61.06 cm^3^ to 33.47 cm^3^ (**Figure 2**). Based on these findings, the patient was ultimately diagnosed with 21-OHD, with the phenotype considered most consistent with the simple virilizing form, which was subsequently genetically supported by the CYP21A2 homozygous mutation (I172N, Exon4) (2, 14). Given the significant short-term adrenal morphological changes and the presence of generalized enlarged lymph nodes, a biopsy of a right cervical lymph node was performed and indicated reactive lymphadenopathy. Additional evaluations were performed to exclude other adrenal-related pathological conditions. Tumor markers were negative, and whole-body PET/CT showed bilateral adrenal thickening with increased glucose metabolism and high somatostatin receptor expression, consistent with adrenal hyperplasia; no other abnormal hypermetabolic lesions suggestive of malignancy were identified. Adrenal hemorrhage was considered unlikely because coagulation parameters were within the normal range and the patient had no history of trauma and anticoagulant use. Infectious etiologies were not supported by normal white blood cell counts and C-reactive protein levels, and tuberculosis antibody testing was negative. Autoimmune-related adrenal involvement was considered unlikely because autoimmune disease-related antibodies were unremarkable. After excluding adrenal-related tumors, hemorrhage, infections, and autoimmune disorders, we speculated that the transient adrenal morphological changes reflected stress-associated compensatory hypertrophy and/or hyperplasia of adrenal cortex in response to infectious stress, aimed at augmenting cortisol secretion to modulate inflammation and maintain homeostasis.

**Figure 2:**
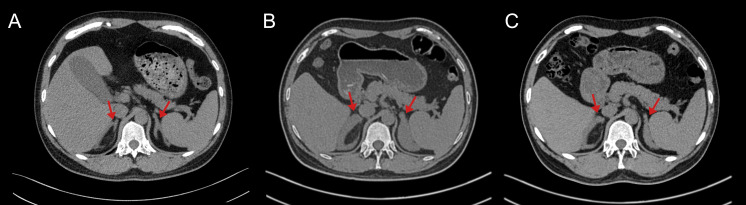
Dynamic changes of adrenal morphology across three time points. **(A)** Axial non-contrast CT obtained 6 years before presentation shows relatively mild bilateral adrenal hyperplasia. **(B)** Axial non-contrast CT during the infectious stress episode demonstrates marked bilateral adrenal enlargement. **(C)** Axial non-contrast CT 1 month after the stress episode shows transient regression of bilateral adrenal enlargement. Red arrows indicate bilateral adrenal enlargement. Adrenal glands volume was calculated semi-automatically by 3D Slicer (Brigham and Women’s Hospital, version 4.11, Harvard University, Boston, MA, USA).

At the time of hospitalization, the patient was clinically stable and had no symptoms suggestive of adrenal crisis or overt adrenal insufficiency. The acute infectious stress had already subsided, and no clinical or biochemical evidence of adrenal crisis was observed during the acute episode. Hormonal evaluation showed basal cortisol remained within the reference range. Therefore, long-term glucocorticoid replacement was not initiated immediately. Instead, close endocrine follow-up and longitudinal monitoring were undertaken. One year later, in the setting of declining cortisol levels and elevated ACTH, oral hydrocortisone acetate (10 mg twice daily) was initiated (**Table 3**). The overall clinical course, imaging studies, endocrine evaluation, and treatment timeline are summarized in **Figure 3**.

**Table 3:** Dynamic changes of cortisol, ACTH, and AGV at different follow-up periods.

Parameter	Initiation of infectious stress	1 month later	2 months later	12 months later
Cortisol (nmol/L)	372	174	199	108
ACTH (ng/L)	44.88	180	84.21	220.1
AGV (cm^3^)	61.03	33.47	34.96	31.20

ACTH, adrenocorticotropic hormone; AGV, adrenal glands volume.

**Figure 3:**
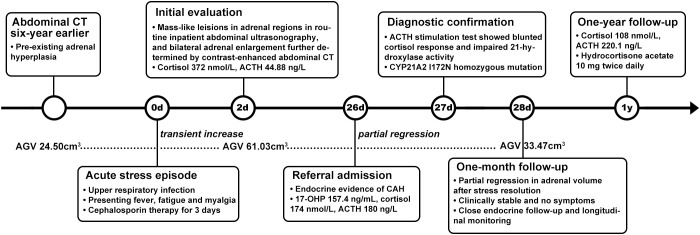
Graphical timeline of the clinical course, imaging studies, endocrine evaluation, and treatment in this patient. Abbreviation: 17-OHP, 17α-hydroxyprogesterone; ACTH, adrenocorticotropic hormone; AGV, adrenal glands volume; CAH, congenital adrenal hyperplasia; CT, computed tomography.

## Discussion

A China study of 7,907 healthy subjects reported a reference of bilateral adrenal glands volume (AGV) as 7.73 cm^3^ (15). In the present case, the patient’s AGV was already increased six years earlier, measuring 24.50 cm^3^ (approximately 3.2-fold higher than the reference value). One month before presentation, following an upper respiratory tract infection, the AGV increased to 61.06 cm³ and subsequently decreased to 33.47 cm³ as the stress subsided.

Patients with 21-OHD have impaired cortisol synthesis in the zona fasciculata due to reduced 21-hydroxylase activity. Insufficient cortisol production weakens negative feedback on the HPA axis, leading to increased ACTH secretion, which in turn promotes adrenal cortical hyperplasia and attempts to maintain cortisol production. In the present case, the endocrine findings are more appropriately interpreted as impaired adrenal reserve rather than overt basal cortisol deficiency. The patient’s residual enzymatic activity may have been sufficient to maintain basal cortisol secretion under non-stressed conditions, but inadequate to mount an appropriate cortisol response during acute stress. This may explain why he remained clinically stable at baseline, while remaining vulnerable to an inadequate stress response, as reflected by transient stress-associated adrenal enlargement and general reactive lymphadenopathy. Therefore, the clinical implication of this case is not generalized cortisol deficiency, but impaired stress adaptability in 21-OHD with residual enzymatic activity, highlighting the importance of comprehensive assessment of adrenal morphology and function and longitudinal endocrine follow-up. Importantly, the decision not to initiate long-term glucocorticoid replacement immediately should not be interpreted as questioning the role of glucocorticoid therapy in patients with simple virilizing CAH due to 21-OHD. In this setting, glucocorticoid therapy is considered not only to prevent adrenal crisis but also to suppress chronic ACTH excess and ACTH-driven androgen overproduction. These considerations were taken into account in the present case. However, given the patient’s stable clinical condition at presentation, relatively preserved basal cortisol production, absence of an immediate clinical indication for urgent androgen suppression, and need for individualized longitudinal assessment, long-term replacement therapy was initially deferred. Hydrocortisone was subsequently initiated when declining cortisol levels with persistently elevated ACTH became evident during follow-up.

As a key effector organ in the stress response, the adrenal gland plays a pivotal role across diverse stressors. During stress, both the HPA axis and the SAMS are activated (16). In response to stress signals, the hypothalamus releases corticotropin-releasing hormone (CRH) and arginine vasopressin, which stimulate the anterior pituitary to secrete ACTH. ACTH then acts on the adrenal cortex to promote glucocorticoid secretion. Through activation of the sympathetic nervous system, the SAMS stimulates the adrenal medulla to produce catecholamines. Additionally, sympathetic activation promotes renin release from juxtaglomerular cells, increasing angiotensin II (AII) production. Both elevated AII and ACTH stimulate aldosterone secretion from the adrenal zona glomerulosa. Furthermore, the adrenal medulla and cortex are interconnected via paracrine signaling and regulation of local blood flow, thereby coordinating adrenal hormone secretion during stress (17–19).

Several animal studies have shown that stress-associated increases in ACTH, can stimulate adrenal cortical cell proliferation, leading to increased adrenal volume and weight (10, 11). Yvonne M et al. (20) reported that chronic stress induced hyperplasia of the outer zona fasciculata in rats, whereas the inner zona fasciculata and medulla exhibited hypertrophy. Upper respiratory tract infection is among the most common triggers of adrenal crisis in patients with CAH (21). In the present case, infectious stress likely activated the HPA axis, and, in the context of impaired cortisol synthesis in CAH with resultant ACTH elevation, contributing to the short-term significant morphological changes of the adrenal glands. This patient also exhibited generalized superficial lymphadenopathy, with biopsy demonstrating reactive hyperplasia, which may reflect inadequate endogenous glucocorticoid production to restrain the inflammatory response. We therefore postulate that the compensatory adrenal enlargement occurred to augment cortisol secretion and mitigate inflammation. Noteworthy, adrenal volume was maximal when the measured ACTH level remained within the reference range, whereas ACTH became markedly elevated only after adrenal volume had substantially decreased. This apparent discordance may be explained, at least in part, by the timing of endocrine assessment. The peak ACTH response may have occurred during the acute infectious phase and therefore was not captured biochemically. Moreover, glucocorticoid-mediated negative feedback on the HPA axis can occur rapidly through nongenomic mechanisms within seconds to minutes, inhibiting CRH and ACTH secretion at the hypothalamic and pituitary levels; delayed genomic feedback may further suppress CRH expression and ACTH synthesis over hours to days (22). Thus, a normal ACTH level at a single time point does not necessarily exclude preceding transient HPA-axis activation. Notably, the cortisol level and adrenal volume showed a more concordant pattern: cortisol was relatively higher when adrenal volume was maximal and subsequently declined as adrenal volume partially regressed. Therefore, the present findings should be interpreted as a dynamic stress-related adrenal response rather than as a simple linear relationship between a single ACTH measurement and adrenal size.

The mechanism underlying adrenal volume reduction after stress resolution also deserves consideration. Once the infectious stress subsided, HPA-axis activation may have decreased, and glucocorticoid-mediated negative feedback may have further attenuated CRH/ACTH drive. Because ACTH has both steroidogenic and trophic effects on the adrenal cortex, reduced ACTH-related stimulation after resolution of stress could allow partial regression of stress-associated cortical hypertrophy and/or hyperplasia (23, 24). In addition, the adrenal cortex is a dynamically remodeling organ; therefore, the observed volume reduction may also reflect decreased cellular hypertrophy, reduced proliferative drive, and tissue remodeling, including apoptosis of adrenocortical cells (25–27). However, due to histological assessment of the adrenal glands was not performed, the precise cellular mechanism of regression remains speculative.

In summary, this case provides an unusual and clinically meaningful demonstration of the tight coupling between adrenal morphology and endocrine dynamics in genotype-confirmed 21-OHD. The marked short-term volume fluctuation following an infectious stressor—characterized by transient enlargement of pre-existing chronic adrenal hyperplasia and subsequent partial regression—together with concomitant changes in ACTH and cortisol, offers rare temporal evidence that adrenal morphology can dynamically change as an adaptive response in 21-OHD with residual enzyme activity. Clinically, this case expands the differential diagnosis of bilateral adrenal lesions and highlights the importance of recognizing 21-OHD beyond childhood. More importantly, it underscores the need for timely and integrated assessment of adrenal morphology and function, including prior and serial imaging, adrenal reserve testing, dynamic hormonal evaluation, and longitudinal endocrine follow-up, particularly when adrenal enlargement occurs in the setting of acute stress.

## Conclusions

This report documents a rare and well-characterized case of transient stress-associated enlargement of pre-existing adrenal hyperplasia in a patient with genetically confirmed 21-OHD. Serial imaging captured a rapid reduction in adrenal volume from 61.06 to 33.47 cm³ within one month. In parallel, endocrine evolution—declining cortisol with rising ACTH and a blunted response to ACTH stimulation—supported limited cortisol reserve despite apparent stress-related compensation. These findings suggest that, in patients with 21-OHD and residual enzymatic activity, infectious stress may precipitate short-term adrenal cortical hypertrophy and/or hyperplasia superimposed on pre-existing chronic adrenal hyperplasia. Clinicians should recognize this pattern when evaluating bilateral adrenal lesions and integrate prior imaging, hormonal assessment, genetic testing, and longitudinal follow-up to reduce the risk of misdiagnosis and support appropriate endocrine management.

## Data Availability

The raw data supporting the conclusions of this article will be made available by the authors, without undue reservation.
